# HETEROGENEITY IN HUMAN IMMUNE AGING AND VACCINE RESPONSES

**DOI:** 10.1093/geroni/igaf122.820

**Published:** 2025-12-31

**Authors:** Duygu Ucar

**Affiliations:** The Jackson Laboratory for Genomic Medicine, Farmington, Connecticut, United States

## Abstract

Aging increases the risk for morbidity and mortality from severe infections and reduces vaccine responses, particularly among older adults. While we know how aging affects immune cells, the heterogeneity among older adults both in terms of immune aging and immune responsiveness to vaccination are less understood. The overarching goal of my laboratory is to uncover the drivers of this heterogeneity, with the ultimate aim of improving immune resilience in older adults. We have identified transcriptomic and epigenomic signatures of immune aging in peripheral blood mononuclear cells (PBMCs) and discovered predictive genomic markers of vaccine responsiveness to pneumococcal and influenza vaccines in older adults. We recently showed that elevated frequencies of cytotoxic CD16+ NK cells in older adults are associated with poor antibody responses to T cell-dependent pneumococcal vaccination, suggesting a novel NK and T helper regulatory axis in immune aging.

